# Transactions of the College of Physicians of Philadelphia

**Published:** 1877-11

**Authors:** 


*Transactions of the College of Physicians of Philadelphia. Volume the Tenth. Third Series, Volume the Third.* Philadelphia: Printed for the College and for sale by Lindsay and Blakiston. 1877.

This volume contains the papers read before the college from October, 1877, to July, 1877, inclusive. These are twelve in number. Case of Trombosis of the Cerebral Veins, by Arthur V. Meigs, M. D. Case of Lacereated wound of Elbow-Joint by Lister's Method, by J. Ewing Mears, M. D. Case of Amputation at Shoulder-joint after necrosis of Humecus and Anchyloses at Shoulder, by J. E. Mears, M. D. The Post-Mortem Imbition of Poisons, by J. J. Reese, M. D. Internal administration of Nitrate of Silver, by Wm. Pepper, A. M., M. D. Case of Malignant Disease, by J. H. Hutchingson, M. D. The State of Medicine in China, R. P. Harris, M. D. Remarks on Relapses in Typhoid Fever, by J. M. Da Costa, M. D. On change in the Nails in fever, by Morris Longstreet, M. D. Report on Meteorology and Epedemic for 1876, by R. A. Cleeman, M. D. Hemiopia and Decussation in the Optic Chiasm, by Geo. C. Harlan, M. D. The Opthalmascope as an aid to Medical Diagnosis, by Morris J. Lewis, M. D. On the treatment of Old Dislocations of the Shoulder by subcutaneous section of the Humerus and formation of a False Joint, J. E. Mears, M. D. We have not the space to review these various articles as they deserve, but can recommend them as instructive and entertaining. C.

